# Buffalo Academy of Medicine

**Published:** 1899-11

**Authors:** Thomas F. Dwyer


### Society Proceedings.

### BUFFALO ACADEMY OF MEDICINE.

REPORTED BY THOMAS F. DWYER, M. D., Secretary.

THE regular quarterly meeting of the Buffalo Academy of Medicine was held at Alumni Hall, University of Buffalo, Tuesday, September 12, 1899.

The president, Dr. MATTHEW D. MANN, called the meeting to order at 8.45 p. m.

The minutes of the last quarterly meeting were read and approved.

Dr. LUCIEN HOWE presented the following resolutions, which on motion were adopted :

WHEREAS, A resolution has been introduced in the Common Council with the view to greatly modify or to repeal the so-called smoke ordinance, and

WHEREAS, While we would be averse to any unnecessary restriction upon manufacturers in our growing city, still as physicians, we appreciate that the requirements of health are above all business considerations, therefore,

*Resolved*, That a committee of three be appointed to represent the Academy of Medicine at the hearings on the proposed ordinance with power to advise for or against any of the changes proposed in this ordinance.

The Chair appointed as such committee, Drs. Lucien Howe, William Warren Potter and P. W. Van Peyma.

Dr. ARTHUR G. BENNETT presented the following request signed by eleven Fellows of the Academy :

We, the undersigned, members of the Academy of Medicine, in good standing, engaged in the practice of ophthalmology, otology, rhinology and laryngology, respectfully request that a section of the Academy be established for the study of the diseases and conditions arising in the eye, ear, nose and throat.

It was moved and seconded that the request be granted.

Dr. PRYOR asked the reason for the proposed new section and what effect it might have on the Academy.

Dr. BENNETT in explaining said that a number of the members, who were specialists in those branches desired to have a separate section and did not see how it could possibly injure the Academy in any way. Dr. Howe predicted a failure for the new section if formed and also said it would lessen the attendance at the surgical section without a resulting benefit.

Dr. GROSVENOR moved that the subject matter be referred to the Council and they be requested to report at the next quarterly meeting. Carried.

The section of medicine entertained the Academy with an interesting discourse by Dr. DANIEL R. BROWER, of Chicago, Ill., on The medical aspects of crime, which was illustrated by stereopticon views.

Dr. BROWER said the progress in science, in literature and in arts had been marvelous during the latter end of the nineteenth century. Medicine and surgery have made mighty strides. The insane, the feeble-minded, the deaf, dumb and blind have received in these later years treatment that is scientific and successful. The care of the criminal is the one great question that baffles society, and great difficulties have arisen from the fact that the medical aspects of crime have not been sufficiently considered.

Lombroso and others, in his opinion, have established the fact that the habitual criminal is an abnormal man, the abnormality manifesting itself (1) physically in the criminal physiognomy, in stigmata of degeneration, in anomalies of the muscular, sensory, respiratory and circulatory systems; and (2) psychically in moral insensibility, lack of forethought, low grade of intelligence, vanity and egoism and emotional instability. Statistics showed that crime is increasing throughout the United States in a vastly more rapid ratio than is the population. The two great etiologic factors in the causation of crime are criminal parentage and environment, and quoted the histories of the Jukes family, the tribe of Ben Ishmael, and the descendents of Frau Ada Jurke.

Another important factor in the cause of crime is intemperance. Alcoholics, it is estimated, are the direct or indirect cause of probably 75 per cent. of all the crime committed. Other factors are the crowding of people together and the raising of children in unhygienic and criminal surroundings, and the criminal laws and their unreasonable execution. The laws are defective, because they are directed against the crime and not the criminal. The study of physiognomy was next mentioned, and is in the opinion of the essayist too much neglected by the physician of the present day. The marks of crime, such as the anomalies of jaw, ear, nose, feet, skin, hair, cranium and brain were thoroughly gone over. The next important aspect is treatment, and as the criminal parentage and environment are the two great causative factors, the treatment should be directed against them. We should prevent propagation by laws regulating marriage and by asexualization. The children of these degenerates should be placed under state control. High license or other methods should be devised to lessen the alcoholic habit. All sentences should be indeterminate; the criminal should be sent to a prison or a reformatory, not for a specific time, but until he is reformed or cured, precisely as a patient is sent to a hospital.

DISCUSSION.

Dr. H. R. Hopkins said the time has come for the medical profession to speak plainly on this subject, and educate the people into the proper consideration of a criminal as one diseased.

Dr. F. E. Fronczak thought we should have a house of detention where persons should be sent to permit them to be examined by medical men. As physician to the penitentiary he had known a number of epileptics to be committed to that institution as drunkards.

Tracy C. Becker, Esq., said the developments of the essayist did not differ much from the experience of most of those who had to do with criminals. The increase of crime in his opinion was due to the influx of degenerates from unrestricted immigration, from indiscriminate marriages and the penal system. He believed that sentences should be indeterminate. Reforms proceed slowly. We had until fifteen or twenty years ago the infamous system of contract labor. This was on the theory that penitentiaries should be self-supporting. It was wiped out principally through the efforts of the labor organisations. The press howled against this and spoke of insanity and the like that would follow criminals remaining idle. Finally, a bill was introduced classifying criminal labor.

Dr. S. A. Dunham believed that the increase in criminal statistics was not due to crime, but rather to arrests for drunkenness, which is not a crime, but a disease.

Dr. J. H. PRYOR disagreed with some statements that had been made. The lawyers are liable to be too conservative, the scientists too radical. There is a danger for statistics in examining criminals with a stated idea. He does not believe that marriage can be controlled by law. He also favored a house of detention where people can be sent without mixing with the worst class of criminals.

Judge Truman C. White said he had pronounced notions about criminal law. He believed the law rather than its administration was defective. In his opinion, the theory of indeterminate sentence is a correct one.

Police Justice Thomas S. King said he was opposed to long sentences, excepting for incorrigible criminals. Leniency in first offences lessens crime. He does not agree with the essayist that crime is decreased by long punishment. He is also in favor of asexualisation for habitual criminals.

Dr. BROWER, in closing, thanked the audience for the close attention given him. This is a radical talk and he was not surprised that some differed from him. He thanked the lawyers for their opinions.

Dr. H. R. HOPKINS moved that a vote of thanks be tendered Dr. Brower for his kindness in coming to Buffalo and reading his interesting paper. Carried.

Attendance, 61. Adjourned at 10.30 p. m.

